# Resting energy expenditure and substrate oxidation rates correlate to temperature and outcome after cardiac arrest - a prospective observational cohort study

**DOI:** 10.1186/s13054-015-0856-2

**Published:** 2015-03-29

**Authors:** Ulrike Holzinger, Richard Brunner, Heidrun Losert, Valentin Fuhrmann, Harald Herkner, Christian Madl, Fritz Sterz, Bruno Schneeweiß

**Affiliations:** Department of Medicine III, Division of Gastroenterology and Hepatology, ICU 13H1, Medical University of Vienna, Waehringer Guertel 18-20, Vienna, 1090 Austria; Department of Emergency Medicine, Medical University of Vienna, Waehringer Guertel 18-20, Vienna, 1090 Austria; Department of Intensive Care, University Medical Center Hamburg-Eppendorf, Martinistraße 52, Hamburg, 20246 Germany

## Abstract

**Introduction:**

Targeted temperature management improves outcome after cardiopulmonary resuscitation. Reduction of resting energy expenditure might be one mode of action. The aim of this study was to correlate resting energy expenditure and substrate oxidation rates with targeted temperature management at 33°C and outcome in patients after cardiac arrest.

**Methods:**

This prospective, observational cohort study was performed at the department of emergency medicine and a medical intensive care unit of a university hospital. Patients after successful cardiopulmonary resuscitation undergoing targeted temperature management at 33°C for 24 hours with subsequent rewarming to 36°C and standardized sedation, analgesic and paralytic medication were included. Indirect calorimetry was performed five times within 48 h after cardiac arrest. Measurements were correlated to outcome with repeated measures ANOVA, linear and logistic regression analysis.

**Results:**

In 25 patients resting energy expenditure decreased 20 (18 to 27) % at 33°C compared to 36°C without differences between outcome groups (favourable vs. unfavourable: 25 (21 to 26) vs. 21 (16 to 26); *P* = 0.5). In contrast to protein oxidation rate (favourable vs. unfavourable: 35 (11 to 68) g/day vs. 39 (7 to 75) g/day, *P* = 0.8) patients with favourable outcome had a significantly higher fat oxidation rate (139 (104 to 171) g/day vs. 117 (70 to 139) g/day, *P* <0.05) and a significantly lower glucose oxidation rate (30 (−34 to 88) g/day vs. 77 (19 to 138) g/day; *P* < 0.05) as compared to patients with unfavourable neurological outcome.

**Conclusions:**

Targeted temperature management at 33°C after cardiac arrest reduces resting energy expenditure by 20% compared to 36°C. Glucose and fat oxidation rates differ significantly between patients with favourable and unfavourable neurological outcome.

**Trial registration:**

Clinicaltrials.gov NCT00500825. Registered 11 July 2007.

**Electronic supplementary material:**

The online version of this article (doi:10.1186/s13054-015-0856-2) contains supplementary material, which is available to authorized users.

## Introduction

Targeted temperature management improves neurological outcome after cardiopulmonary resuscitation (CPR) although the target temperature is matter of discussion [1-3]. According to the guidelines of the European Resuscitation Council and the American Heart Association comatose patients resuscitated after cardiac arrest should undergo therapeutic hypothermia with a target temperature of 32 to 34°C for 24 hours [4,5].

Reduction of resting energy expenditure (REE) might be one of the possible mechanisms underlying the protective effects of hypothermia provided that the counterregulatory mechanism of shivering is prevented by the use of adequate medication [6,7]. The extent of REE reduction is expected to be approximately 8% per °C [6,7]. In critically ill patients with fever, indirect calorimetry showed REE reduction by 6 to 12% per °C using external cooling [8,9]. In patients with traumatic brain injury no reduction of REE was found below 35°C in contrast to temperatures higher than 35°C [10].

Studies evaluating the influence of hypothermia on substrate metabolism are rare. Most of them are animal investigations or studies including non-sedated humans and can, therefore, not be compared to the patients in the present study [11-14].

Influence of therapeutic hypothermia on REE and substrate metabolism in patients resuscitated after cardiac arrest has not been investigated so far. Therefore, the objective of this study was the investigation of the correlation of REE and substrate oxidation rates with temperature management at 33°C after cardiac arrest and outcome.

## Methods

This was a prospective, observational study using indirect calorimetric assessments in patients undergoing targeted temperature management at 33°C after cardiac arrest as part of routine clinical care according to established protocols in our Department of Emergency Medicine [15]. The study was investigator-initiated and investigator-driven. The study protocol conformed to the ethical guidelines of the Declaration of Helsinki and was approved by the research ethics committee of the Medical University of Vienna. According to the Austrian law the Institutional Review Board for human studies approved the protocol with an exception from informed consent guidelines. None of the patients ended up having consent refused by next of kin or recovered patients after they awoke and were contacted.

The study was performed in the intensive care units (ICUs) of the Department of Medicine III, Division of Gastroenterology and Hepatology (Intensive Care Unit 13 h1) and the Department of Emergency Medicine at the Medical University Hospital of Vienna.

Patients were eligible for inclusion in the study if they were older than 18 years of age and hospitalized within 6 hours after resuscitation from cardiac arrest.

### Treatment

#### General

Patients were stabilized according to the treatment protocols [15]. Standards of post-resuscitation care included airway management and mechanical ventilation, treatment of haemodynamic instabilities and arrhythmias, blood glucose control and temperature control with a temperature probe in the oesophagus. For every patient, age, sex, presumed cause of cardiac arrest, initial rhythm, duration of CPR, comorbidities as well as laboratory data and medication were documented. Patients were followed up at 1, 6 and 12 months according to the Utstein Style using the following scoring instruments: Glasgow coma scale (GCS), cerebral performance category (CPC), and overall performance category (OPC) [16].

### Targeted temperature management

All patients were cooled using either Alsius Coolgard 3000™ (Zoll Medical Corporation., Chelmsford, MA, USA) or Arctic Sun™ (Medivance, Inc., Louisville, CO, USA) with the maximal cooling rate until the target core temperature of 33°C. The cooling period lasted 24 hours and rewarming was performed with a rate of 0.4°C/hour until a core temperature of 36°C was reached.

### Medication

All patients received sedation and analgesia with midazolam and fentanyl as well as muscle paralysis with rocuronium according to the therapeutic standards at the Department of Emergency Medicine at the Medical University of Vienna [15]. Depth of neuromuscular blockade was assessed using the TOF Watch™ SX (Organon Medical Systems, Roseland, NJ, USA), which monitors neuromuscular transmission during surgery or intensive care by means of acceleromyography. Two electrodes are placed above the ulnar nerve and the response to the nerve stimulation is measured by using a small piezoelectrode acceleration transducer distally placed on the volar side of the thumb. Four pulses are given and the measuring is based upon a ratio of the amplitude of the fourth evoked mechanical response to the first one. Sedation as well as analgesic and paralytic medication was stopped after rewarming at 36°C. Patients did not receive any intravenous fluids containing dextrose during the study period. As preferred, crystalloid Ringer lactate solution was used. Some patients received isotonic saline solution. Medications were dissolved or diluted using isotonic saline or Ringer lactate solution or distilled water as specified.

### Indirect calorimetry

Respiratory gas exchange was measured by computerized open-circuit indirect calorimetry (Deltatrac™ II Metabolic Monitor, Datex-Ohmeda Instruments, Helsinki, Finland) as previously described [17,18]. Oxygen consumption and carbon dioxide production were measured in 1-minute intervals and the average of a 30-minute period was calculated. The paramagnetic oxygen sensor allows inspiratory oxygen concentration up to 65% according to the manufacturer. Measurements were performed over a period of 1 hour. Data for further calculation were taken from the second half of the measurement to assure steady state conditions. Ventilator setting remained unchanged at least half an hour before and during measurement. Patients did not receive any form of caloric intake during the cooling and rewarming period. They underwent an 8-hour fasting period before the last calorimetry. To get an overview about REE and substrate metabolism in the different phases of post-resuscitation care (stable cooling phase, passive rewarming, active rewarming, rewarmed stable phase) indirect calorimetry was performed five times: 12 to 24 hours after achieving the target temperature of 33°C (stable phase), during rewarming at a temperature of 34.5°C, 36°C, 36.5 to 37.5°C and 48 hours after cardiac arrest. Sedation as well as analgesic and paralytic medication was stopped after the measurement at 36°C.

### Calculations

REE was expressed in kJ (Kcal)/day/m^2^ body surface area. REE and oxidation rates for glucose, fat, and protein were calculated according to Ferrannini *et al*. [19]. It was assumed that for each 1 g nitrogen produced, 5.923 L oxygen were consumed and 4.754 L carbon dioxide were produced (respiratory quotient for protein: 0.803) [20]. For calculation of urea nitrogen appearance rates, changes in plasma urea concentration were taken into account (measurement at the beginning and at the end of each calorimetry) [21]. Urine production was measured along with each calorimetric measurement. Urinary urea nitrogen was measured colorimetrically [22]. The protein oxidation rate (g/d) was calculated as 6.25 × 24-hour urea nitrogen production (g/d) [23].

### Statistical analysis

Due to the pilot study character and data characteristics we regarded a sample size of 25 patients to be feasible and adequate to yielding sufficient precision of our estimates. Continuous data are presented as median and 25 to 75% interquartile range (IQR) or mean ± standard deviation as appropriate by distribution. Categorical data are presented as count and relative frequency. Twenty-five independent patients each contributed data from five occasions defined by certain temperature levels. Accordingly, we treated data as panel data. Generally linear effects were assessed across categories of temperature and not across actual temperature on a °C scale.

To assess the influence of temperature on metabolic variables we used linear random intercept models. The independent variable was the metabolic variable, temperature was modelled as linear variable, and the clustering variable was a patient identifier. To assess a potential relation between metabolic variables and sedation we grouped data according to temperature ≤36°C and >36°C, because sedation protocols were linked to core temperature. We then repeated the above regression by replacing temperature by the dichotomous variable sedation.

To assess a potential association between metabolic variables and neurological outcome we calculated temperature level-wise independent *t* tests adjusted for multiplicity by the Bonferroni method. We then developed a logistic random intercept model where favourable neurological outcome was the independent variable, and each one of the metabolic variables was entered as predictor, whilst allowing for the panel structure of the data.

To assess the influence of norepinephrine dose on metabolic variables we used linear random intercept regression models with each metabolic variable as independent variable, and norepinephrine therapy as predictor allowing for clustering as described above. Likewise, we investigated the influence of temperature on insulin dose (U/hour) and serum creatinine (mg/dl). Both variables were handled as continuous outcomes. We also tested for an interaction of norepinephrine on the relation between neurological outcome and metabolic variables.

To reassess our models we employed standard regression models using robust standard errors. Results from both approaches were comparable for all calculations. For data management we used Excel 2008 for Mac (Microsoft Corporation, Redmond, WA, USA), for data analysis we used Stata 9.0 for Mac (Stata Corp, College Station, TX, USA). Generally a two-sided *P* value less than 0.05 was considered statistically significant.

## Results

Patient characteristics and outcome are given in Table 1. The median sequential organ failure assessment (SOFA) score was 10 (9.25 to 10.75) in patients with unfavourable neurological outcome and 10 (9 to 11) in patients with favourable neurological outcome. Targeted temperature management was performed using surface cooling in 15 patients and intravascular cooling in 10 patients. We compared C-reactive protein, fibrinogen and leukocyte count as markers of inflammation as well as platelet count, D-dimer, prothrombin time and activated partial thromboplastin time as markers of coagulation between the patient groups undergoing different cooling methods. No differences were observed between the two different cooling methods (data not shown). Mean train-of-four (TOF) value was 0. 114 indirect calorimetric measurements were performed. Two patients died after the first measurement and in one patient indirect calorimetry had to be stopped after the third measurement due to technical failure of the monitor. In one patient the last measurement could not be performed because of a fraction of inspired oxygen (FiO_2_) level of 100% due to adult respiratory distress syndrome after aspiration. In case of premature study termination collected data were used for analysis until the patient left the study.

**Table 1:** **Patients’ characteristics and outcome data (n = 25)**

**Age (years)**	**61 ± 14**
BMI (kg/m^2^)	26 ± 4
BSA (m^2^)	1.9 ± 0.2
Female – no (%)	6 (24)
Reason for CA – no (%)	
MCI	15 (60)
Rhythm disorder	6 (24)
Others	4 (16)
Initial cardiac rhythm – no (%)	
VF/VT	21 (84)
Asystole/PEA	4 (16)
CA to ROSC (min)	25 (14-32)
Neurological outcome at 12 months – no (%)	
CPC 1–2 (favourable)	15 (60)
CPC 3–5 (unfavourable)	10 (40)
Survival – no (%)	16 (64)

Values are expressed as absolute numbers and percentage and as mean value ± standard deviation or median with 25 to 75% quartiles. Others comprise one intoxication with consecutive apnea, one high-voltage accident, one pulmonary embolism and one unknown reason. BMI, body mass index; BSA, body surface area; CA, cardiac arrest; MCI, myocardial infarction; VF, ventricular fibrillation; VT, ventricular tachycardia; PEA, pulseless electric activity; ROSC, return of spontaneous circulation; CPC, cerebral performance category.

**Figure 1:**
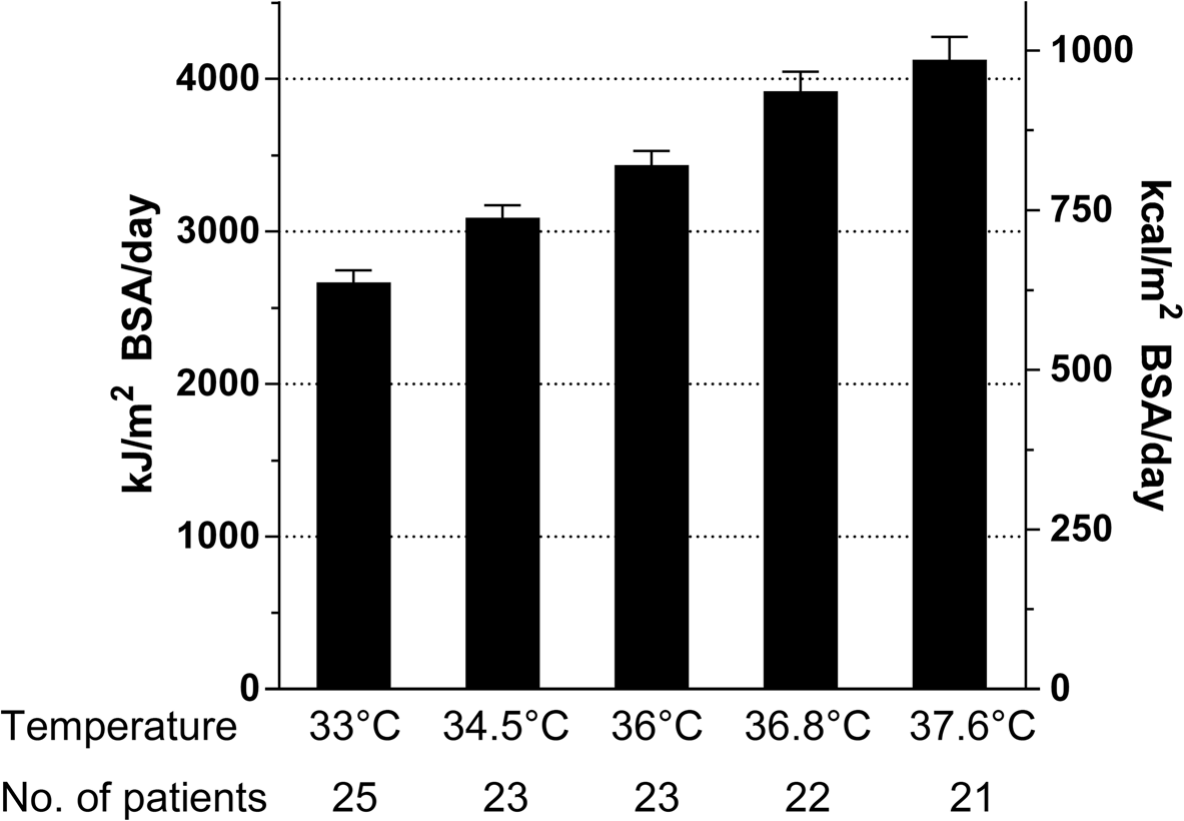
**Resting energy expenditure (REE) of all patients measured with indirect calorimetry at the different temperatures.** REE is given in kJ/m^2^ (kcal/m^2^) of body surface area (BSA)/day. A linear relation between REE and temperature was detected (297 kJ (71 kcal)/m2/°C category; *P* <0.0001).

**Figure 2:**
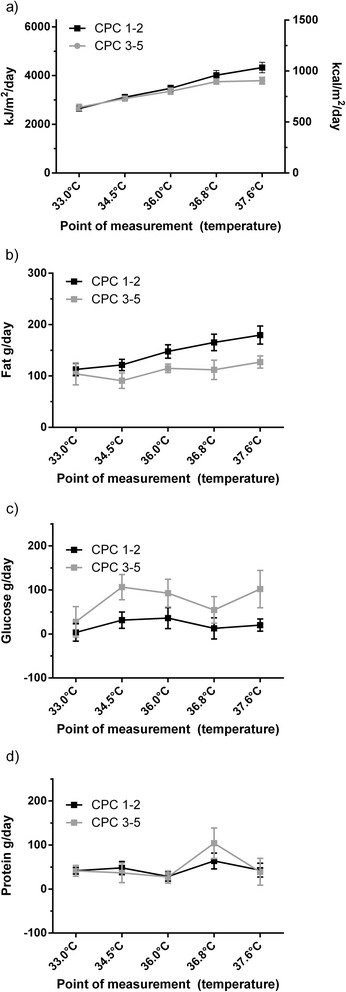
**Changes in metabolic variables during the whole study period assessed with indirect calorimetry at the defined time points.** Comparison of patients with favourable neurological outcome (CPC 1 to 2) with unfavourable neurological outcome (CPC 3 to 5): black dots ●, favourable neurological outcome; grey squares ■, unfavourable neurological outcome, error bars indicate standard deviation. **(a)** Linear regression of REE did not show a difference between patients with favourable and unfavourable neurological outcome (*P* = 0.2). **(b)** Linear regression of fat oxidation rates revealed a significant difference between patients with favourable and unfavourable neurological outcome (*P* <0.05). **(c)** Linear regression of glucose oxidation rates revealed a significant difference between patients with favourable and unfavourable neurological outcome (*P* <0.05). **(d)** Linear regression of protein oxidation rates did not show a difference between patients with favourable and unfavourable neurological outcome (*P* = 0.8). CPC, cerebral performance category; REE, resting energy expenditure.

Time points of indirect calorimetric measurements resulted in temperature categories of 1.5°C below 36°C and temperature categories of 0.8°C above 36°C (as presented in Figures 1 and 2 and Table 2). The overall mean oxygen consumption (VO_2_) was 241 ml/min with a range from 125 to 662 ml/min over all measurements. Within individuals the mean standard deviation (SD) was 13 ml/min (5% of the mean) representing measurement variability. Between individuals, SD was 68 ml/min on average (28% of the mean) representing the variability between the patients. During the initial stable conditions (with sedation, analgesics and paralysis at 33°C) variability was smaller: overall range 125 to 476 ml/min. Within-individual SD was 9.00 ml/min (4.5% of the mean). The between-individual SD was 52.13 ml/min (26% of the mean).

**Table 2:** **Substrate metabolism of all patients at different calorimetric measurement**

	**Points of measurement (°C)**				
	**33**	**34.5**	**36**	**36.8**	**37.6**
RQ	0.72 (0.69-0.83)	0.76 (0.72-0.82)	0.76 (0.74-0.79)	0.75 (0.72-0.79)	0.75 (0.73-0.78)
VO2 (ml/min)	187 (147-220)	215 (179-248)	243 (191-266)	264 (221-313)	259 (246-311)
VCO_2_ (ml/min)	136 (122-151)	162 (138-187)	185 (148-199)	193 (173-232)	198 (189-234)
pH	7.36 (7.29-7.41)	7.36 (7.31-7.40)	7.37 (7.32-7.40)	7.35 (7.26-7.40)	7.34 (7.29-7.43)
Lactate (mmol/l)	1.4 (1.0-2.0)	1.4 (1.0-1.9)	1.2 (0.9-1.7)	1.1 (0.9-1.4)	1.0 (0.9-1.6)
UNP (g/d)	6.1 (2.3-9.1)	4.2 (1.2-12.1)	3.4 (1.9-8.8)	9.7 (4.7-22.1)	4.4 (0.5-16.3)
Glucose (g/d)	−5 (−50-92)	79 (−13-113)	67 (14-106)	36 (−27-86)	50 (5-105)
Fat (g/d)	125 (55-154)	118 (69-137)	128 (109-164)	153 (106-175)	140 (119-178)
Protein (g/d)	38.3 (14.2-57.1)	25.9 (7.7-75.3)	21.4 (11.9-55.3)	60.4 (29.6-137.8)	27.5 (2.9-101.9)
Creatinine (mg/dl)	0.96 (0.65-1.05)	0.87 (0.70-0.99)	0.92 (0.79-1.05)	0.91 (0.73-1.07)	0.98 (0.76-1.14)

Negative glucose values reflect net gluconeogenesis whereas positive values indicate glucose oxidation. Additionally arterial blood ph and lactate are given as marker of aerobe metabolism and creatinine as marker of renal function. Values are expressed as median with 25 to 75% quartiles. RQ, respiratory quotient; VO_2_, oxygen consumption; VCO_2_, carbon dioxide production; UNP, urea nitrogen production.

Calorimetric measurements and substrate metabolism of all patients are given in Table 2. Negative glucose values reflect net gluconeogenesis whereas positive values indicate glucose oxidation.

REE showed a reduction of 20 (18 to 27) % at 33°C compared to 36°C without differences between outcome groups (favourable vs. unfavourable: 25 (21 to 26) vs. 21 (16 to 26); *P* = 0.5) and a linear relation to temperature alterations (297 kJ (71 kcal)/m^2^/°C category; *P* <0.0001) (Figure 1).

Fat oxidation rate showed a temperature dependency (10 g/day/°C category; *P* <0.0001), while glucose and protein oxidation rates were not significantly dependent from temperature (*P* = 0.07 or *P* = 0.06, respectively).

In contrast to protein oxidation rate (favourable vs. unfavourable outcome: 35 (11 to 68) g/day vs. 39 (7 to 75) g/day, *P* = 0.8) patients with favourable outcome had a significantly higher fat oxidation rate (139 (104 to 171) g/day vs. 117 (70 to 139) g/day, *P* <0.05) and a significantly lower glucose oxidation rate (30 (−34 to 88) g/day vs. 77 (19 to 138) g/day; *P* <0.05) as compared to patients with unfavourable neurological outcome (Figure 2).

REE was not associated with neurological outcome (odds ratio (OR) 0.88, 95% confidence interval (CI) 0.63 to 1.22 of favourable neurological outcome for each unit increase in REE quartile, *P* = 0.4; Figure 2). Probability of unfavourable neurological outcome increased by 69% (OR 1.69, 95% CI 1.15 to 2.49; *P* <0.01) with every increase of glucose oxidation rate from one quartile to the next. Probability of unfavourable neurological outcome decreased by 38% (OR 0.62, 95% CI 0.44 to 0.87; *P* <0.01) with every increase of fat oxidation rate from one quartile to the next.

For all patients dosage of sedation and analgesic medication (midazolam, fentanyl), neuromuscular blocker (rocuronium) and norepinephrine as well as insulin at the different measurement points is given in the electronic supplementary file (Table S1 in Additional file 1). One patient received dobutamine during the rewarming period (measurement 2, 3 and 4). Another patient received levosimendan during the first two measurements. Although both medications might influence metabolism, subgroup analysis was not possible due to the small number of patients.

Insulin therapy was not associated with outcome (*P* = 0.3), REE (*P* = 0.7) or substrate oxidation rates (glucose: *P* = 0.2; fat: *P* = 0.9; protein: *P* = 0.7).

Sedation, analgesia and neuromuscular blockade (NMB) rates were strongly associated with REE reduction; (967 kJ (231 kcal)/day/m^2^ (95% CI 181 (758) to 280 (1,172); *P* <0.0001). Likewise fat oxidation rate was reduced by 34 g/d (95% CI 14 to 54; *P* <0.01) during sedation. Glucose oxidation rate (*P* = 0.9) and protein oxidation (*P* = 0.06) were not significantly associated with sedation.

Even though we did not find an association between norepinephrine therapy and REE (*P* = 0.7) or protein oxidation rate (*P* = 0.6), norepinephrine therapy was associated with increased glucose oxidation rate (*P* <0.05) and reduced fat oxidation rate (*P* <0.05). However, there was no significant interaction of norepinephrine on the relation between neurological outcome and metabolic variables. Norepinephrine therapy was not associated with outcome (*P* = 0.7).

Temperature was not associated with insulin therapy (*P* = 0.6) or serum creatinine levels (*P* = 0.8). We did not find an association between outcome and serum creatinine levels (*P* = 0.6).

Dosage of all administered medications and blood glucose values at different calorimetric measurements are given in the Table S1 in Additional file 1 for all patients.

## Discussion

Our study showed that in patients after cardiac arrest and successful return of spontaneous circulation undergoing targeted temperature management at 33°C REE was reduced (20% (18 to 27%)) compared to 36°C with a linear relation to temperature alterations but without differences between outcome groups. Besides temperature, sedation, analgesia and NMB were associated with a REE reduction.

Only fat oxidation rate was associated with temperature, sedation and analgesia. Glucose oxidation rates and fat oxidation rates were significantly different between patients with favourable and unfavourable neurological outcome. An interaction between norepinephrine therapy, neurological outcome and metabolic variables could be excluded. Use of medication (sedatives, analgetics, neuromuscular blockers, insulin and norepinephrine) was not different between patients with favourable and unfavourable neurological outcome.

The effect of cooling on REE has already been described. In hyperthermic patients cooling was able to reduce REE between 6 and 12% per 1°C temperature reduction depending on sedation, analgesia and NMB [7,8]. In neurosurgical patients, a significant reduction of REE by cooling was only detectable above a temperature of 35°C using sedation and neuromuscular blockers [10]. Generally, a reduction of 6 to 9% of REE per 1°C decrease in temperature is accepted [6,7]. Our study showed a 6.6% reduction of REE per 1°C decrease at temperatures below 36°C.

A limitation of our study is that effects of sedation, analgesia, NMB and hypothermia cannot be separated due to the study design. All indirect calorimetric measurements at 36°C and below were performed with concomitant sedation, analgesia and NMB.

In surgical ICU patients increasing Ramsay sedation scale using midazolam resulted in a significantly decreased REE and oxygen consumption [24]. NMB in combination with controlled mechanical ventilation was able to reduce oxygen consumption by approximately 20% [25]. In critically ill children NMB was able to reduce REE by approximately 10% [26]. We found a median reduction of REE of 20% in our study in adult patients after cardiac arrest at 33°C. Our data suggest that effects of sedation, analgesia, NMB and hypothermia on REE might not be additive. This assumption is supported by another study, which showed that in normothermic patients sedation has a major effect on REE; however, in sedated patients temperature was the main determinant of REE [27].

Due to the chosen medication regimen with continuous midazolam, fentanyl and rocuronium administration during the cooling and the passive rewarming period the effect of shivering, which is known to highly influence REE, can be disregarded [28,29].

In our study, patients after cardiac arrest and return of spontaneous circulation (ROSC) with favourable neurological outcome had significantly different fat and glucose oxidation rates compared to patients with unfavourable neurological outcome. An increase in quartiles of fat oxidation rate was associated with favourable and an increase in quartiles of glucose oxidation was associated with unfavourable neurological outcome.

Data on substrate metabolism during hypoxia and hypothermia were only available from animal experiments or experiments with healthy subjects. In six healthy males resting at a temperature of 5°C, free fatty acid turnover, as well as lipid oxidation and carbohydrate oxidation remarkably increased, however this increase was accompanied by a significant increase in the REE [11]. The increase of REE in this experiment is predominantly caused by shivering and thus is not comparable to our analysis.

Hypoxia seems to be associated with remarkable changes in substrate utilization especially in the brain. In cooled anaesthesized dogs, hypoxic conditions increased glucose utilization in the brain [12]. Further animal experiments showed that glucose transporter (GLUT) 3 expression increases up to nine times both in the affected and non-affected neurons after 48 hours of rat brains under ischaemic conditions [13]. This finding is in accordance with data from a rat model of traumatic brain injury, which showed a 300% increase of GLUT 3 expression up to 48 hours after the event [14]. In traumatic brain injury, a remarkable increase in glucose utilization of the whole brain (hyperglycolysis) can be found up to 7 days after the trauma [30,31]. We therefore hypothesize that in patients with unfavourable neurological outcome increased cerebral glucose utilization was present due to severe hypoxia-induced brain injury during cardiac arrest. We assume that this increase accounted for the elevated glucose oxidation rates measured by indirect calorimetry. Since brain glucose utilization normally accounts for 25% of whole body glucose metabolism [32], changes in glucose oxidation rates of the brain are obviously detectable by indirect calorimetry evaluating whole body substrate metabolism. We cannot rule out influence of other post-cardiac arrest organ injury, especially liver and kidney, on REE and substrate metabolism in our study patients [33,34]. However apart from brain injury, other organ injury seemed to be equally distributed in both patient groups and, therefore, is improbable of having caused the effect shown in our study.

Norepinephrine therapy was significantly associated with glucose and fat oxidation rates but did not interact with the relation between neurological outcome and metabolic values and was not associated with outcome in the present study. Influence of norepinephrine on fat utilization has already been shown before in healthy subjects [35].

Although hypothermia is associated with increased insulin resistance and glucose intolerance, insulin therapy was not associated with temperature in our study [6,36]. Insulin was neither associated with REE nor with substrate oxidation rates. Influence of insulin on substrate oxidation rates and energy expenditure seems to be limited [37,38].

A further limitation of our study is the technique of indirect calorimetry itself, which has the advantage of being non-invasive, however, is prone to multiple errors. Although, measurements were performed according to the manufacturer’s instructions and with greatest diligence, errors influencing our results cannot be fully excluded [39]. Using indirect calorimetry, only whole body net balances of substrate metabolism can be described without knowledge of localized substrate oxidation or biosynthesis rates. We can rule out bias of anaerobic metabolic pathways in a substantial way because lactate levels were in the normal range throughout the study period. That is why measurements started in the second half of the 24-hour cooling period, in order to have already achieved stabilized conditions, without anaerobic metabolism.

Besides the small number of patients included, a major limitation of this study is the substantial variability of the measurements of substrate metabolism themselves. The factors that impact substrate metabolism - including the specific blend of organ failures, host global and organ-specific inflammation and function - are multitudinous. Accordingly, we cannot discount the possibility of confounding by variables not measured in this study like interleukin levels or organ-specific substrate metabolism, but our study nonetheless raises an interesting hypothesis for future studies that might control for the most important confounders. Results have to be reproduced and further investigation is necessary to elucidate pathophysiological actions during the post-cardiac arrest period resulting in detectable metabolic differences between patients with favourable and unfavourable neurological outcome.

## Conclusions

Targeted temperature management at 33°C after cardiac arrest reduces REE by 20% with a linear relation to temperature variations. Only fat oxidation rate was temperature dependent. A significant difference in glucose and fat oxidation rates was found between patients with favourable and unfavourable neurological outcome. An increase of fat oxidation rate and a decrease of glucose oxidation rate were associated with favourable outcome.

## Key messages

- Resting energy expenditure is reduced in patients undergoing targeted temperature management after cardiac arrest irrespective of outcome.
- A difference in fat and glucose oxidation rates can be found between patients with favourable and unfavourable neurological outcome.

## Additional file

Additional file 1:
**Mean dosage of medications given to patients with favourable and unfavourable neurological outcome and mean blood glucose at different measurements.** All medications are given as median and interquartile range. Blood glucose is given as mean ± SD.

## Abbreviations

BMI: body mass index
BSA: body surface area
CI: confidence interval
CPC: Cerebral Performance Category
CPR: cardiopulmonary resuscitation
FiO_2_: fraction of inspired oxygen
GCS: Glasgow coma scale
GLUT: glucose transporter
ICU: intensive care unit
IQR: interquartile range
MCI: myocardial infarction
NMB: neuromuscular blockade
OPC: overall performance category
OR: odds ratio
PEA: pulseless electric activity
REE: resting energy expenditure
ROSC: return of spontaneous circulation
RQ: respiratory quotient
SD: standard deviation
SOFA score: sequential organ failure assessment score
TOF: train-of-four
UNP: urea nitrogen production
VCO_2_: carbon dioxide production
VF: ventricular fibrillation
VO_2_: oxygen consumption
VT: ventricular tachycardia
